# Orthoform in the Diagnosis of Gastric Ulcer

**Published:** 1904-10-29

**Authors:** 


### ORTHOFORM IN THE DIAGNOSIS OF GASTRIC ULCER.

On the ground that orthoform has no anæsthetic action on surfaces which are protected by skin or mucous membrane, Dr. F. H. Murdoch[1] recommends its use for diagnostic purposes in cases of gastric pain where an ulcer is suspected. He argues that if the administration of orthoform is speedily followed by relief from the pain, it is strong evidence that there is a breach of surface where nerve endings are exposed to irritation by the contents of the stomach. In support of his opinion he mentions three cases where the method proved of service. In one, appendicitis had been thought to be the cause of the pain; in another, biliary colic had been diagnosed; and in the third, the symptoms had been attributed to gastritis. It is noteworthy that in the first and third cases free hydrochloric was not found in the stomach contents. In each instance pain ceased within half an hour of giving 8 grs. of orthoform.

[1] Med. News, Oct. 8, 1904.

